# Genome-Wide Localization Study of Yeast Pex11 Identifies Peroxisome–Mitochondria Interactions through the ERMES Complex

**DOI:** 10.1016/j.jmb.2015.03.004

**Published:** 2015-06-05

**Authors:** M. Mattiazzi Ušaj, M. Brložnik, P. Kaferle, M. Žitnik, H. Wolinski, F. Leitner, S.D. Kohlwein, B. Zupan, U. Petrovič

**Affiliations:** 1 Department of Molecular and Biomedical Sciences, Jožef Stefan Institute, Jamova 39, SI-1000 Ljubljana, Slovenia; 2 Faculty of Computer and Information Science, University of Ljubljana, Večna pot 113, SI-1000 Ljubljana, Slovenia; 3 Institute of Molecular Biosciences, University of Graz, BioTechMed Graz, Humboldtstrasse 50, A-8010 Graz, Austria; 4 Department of Molecular and Human Genetics, Baylor College of Medicine, 1 Baylor Plaza, Houston, TX 77030, USA

**Keywords:** PPAR, peroxisome proliferator-activated receptor, ER, endoplasmic reticulum, GFP, green fluorescent protein, MYTH, membrane yeast two-hybrid, BiFC, bimolecular fluorescence complementation, high-content microscopy, computational image analysis, lipid metabolism, peroxisomal disorders, organelle juxtaposition

## Abstract

Pex11 is a peroxin that regulates the number of peroxisomes in eukaryotic cells. Recently, it was found that a mutation in one of the three mammalian paralogs, PEX11β, results in a neurological disorder. The molecular function of Pex11, however, is not known. *Saccharomyces cerevisiae* Pex11 has been shown to recruit to peroxisomes the mitochondrial fission machinery, thus enabling proliferation of peroxisomes. This process is essential for efficient fatty acid β-oxidation. In this study, we used high-content microscopy on a genome-wide scale to determine the subcellular localization pattern of yeast Pex11 in all non-essential gene deletion mutants, as well as in temperature-sensitive essential gene mutants. Pex11 localization and morphology of peroxisomes was profoundly affected by mutations in 104 different genes that were functionally classified. A group of genes encompassing *MDM10*, *MDM12* and *MDM34* that encode the mitochondrial and cytosolic components of the ERMES complex was analyzed in greater detail. Deletion of these genes caused a specifically altered Pex11 localization pattern, whereas deletion of *MMM1*, the gene encoding the fourth, endoplasmic-reticulum-associated component of the complex, did not result in an altered Pex11 localization or peroxisome morphology phenotype. Moreover, we found that Pex11 and Mdm34 physically interact and that Pex11 plays a role in establishing the contact sites between peroxisomes and mitochondria through the ERMES complex. Based on these results, we propose that the mitochondrial/cytosolic components of the ERMES complex establish a direct interaction between mitochondria and peroxisomes through Pex11.

## Highlights

• Molecular function of Pex11, a protein with roles in metabolism and disease, is unknown.
• Genome-wide screening determined subcellular localization of Pex11-GFP in yeast.
• Mutants defective in components of the ERMES complex show altered Pex11 localization.
• Pex11 physically interacts with the ERMES complex component Mdm34.
• ERMES complex and Pex11 mediate interaction between mitochondria and peroxisomes.

## Graphical abstract

**Figure:**
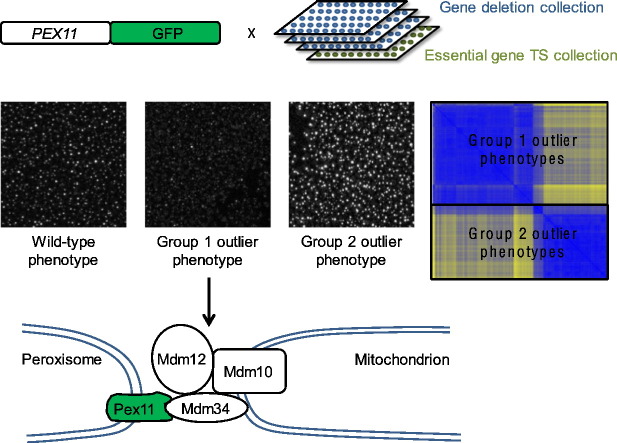

## Introduction

Peroxisomes are ubiquitous yet metabolically diverse organelles whose most common activities are fatty acid β-oxidation and detoxification of reactive oxygen species, especially H_2_O_2_ [1]. Their importance in humans is demonstrated through severe effects of peroxisomal disorders, such as the Zellweger syndrome [2]. In animals, β-oxidation of fatty acids takes place predominantly in mitochondria, except for long-chain fatty acids that are oxidized in peroxisomes, which gain importance also when the animal is fed a high-fat diet, or following pharmacological activation of peroxisome proliferator-activated receptors (PPARs) with drugs such as fibrates [3].

In contrast, in yeast *Saccharomyces cerevisiae*, fatty acid β-oxidation takes place exclusively in peroxisomes. When yeast cells are grown on glucose, there are one or few peroxisomes per cell that exhibit only low basal activity of fatty acid β-oxidation [4]. Fatty acid β-oxidation and proliferation of peroxisomes are triggered in yeast cells after exposure to fatty acids such as oleic acid. In this process, numerous new organelles are produced from pre-existing peroxisomes. When fatty acids are depleted from the medium or after re-introduction of glucose, peroxisomes are degraded via the vacuole by pexophagy [5].

Pex11 is the peroxin (i.e., a protein involved in biogenesis or organization of peroxisomes) whose activity is required for the tubulation and fission of the single peroxisomal membrane during peroxisome proliferation. It is the most highly expressed peroxin under peroxisome proliferation non-inducing conditions and is the most abundant peroxisomal membrane protein [6,7]. Pex11 was proposed to increase membrane curvature, initiating tubulation [8], and was also shown to recruit the mitochondrial fission machinery, which is responsible also for the fission of newly forming peroxisomes [9,10]. In addition, yeast Pex11 was proposed to be a predecessor of the ligand binding domain of metazoan and also mammalian nuclear receptors, most notably PPARs [11], but no related molecular function to nuclear receptors has been found for Pex11. Notably, mutations in the *PEX11* genes can lead to diseases in mammals: deficiency in PEX11β, one of the three mammalian paralogs of Pex11, causes neurological and developmental defects of the Zellweger syndrome spectrum in mice [12,13], and a mutation in *PEX11*β was found in a patient with symptoms somewhat atypical for peroxisome biogenesis disorders [14].

The proposed functional relation of the yeast Pex11 to PPARs is intriguing as Pex11 is a membrane-bound protein, in contrast to the soluble nuclear receptors [15]. It is also not understood how mutations in human *PEX11*β lead to disease. In fact, despite several biological processes being linked to Pex11, the molecular function of this protein remains unknown. To elucidate the factors controlling localization and abundance of Pex11 in yeast cells, we conducted a genome-wide imaging-based screen of Pex11 localization. This analysis allowed us to identify such cellular factors and provided insight into critical Pex11-associated cellular processes.

## Results

### Genome-wide screen of Pex11 localization

We used high-content microscopy on a genome-wide scale to accurately determine the subcellular localization pattern of yeast Pex11 in all non-essential gene deletion mutants and in temperature-sensitive essential gene mutants. Pex11 was genetically tagged with green fluorescent protein (GFP) in the library of 4292 non-essential gene deletion strains and 793 strains with temperature-sensitive alleles of 503 essential genes. Fluorescence microscopy was performed using a confocal microscope to screen the whole collection in matter of days, as previously described [16]. All the images are available in the online image database *YPLpex*||.

**Fig. 1:**
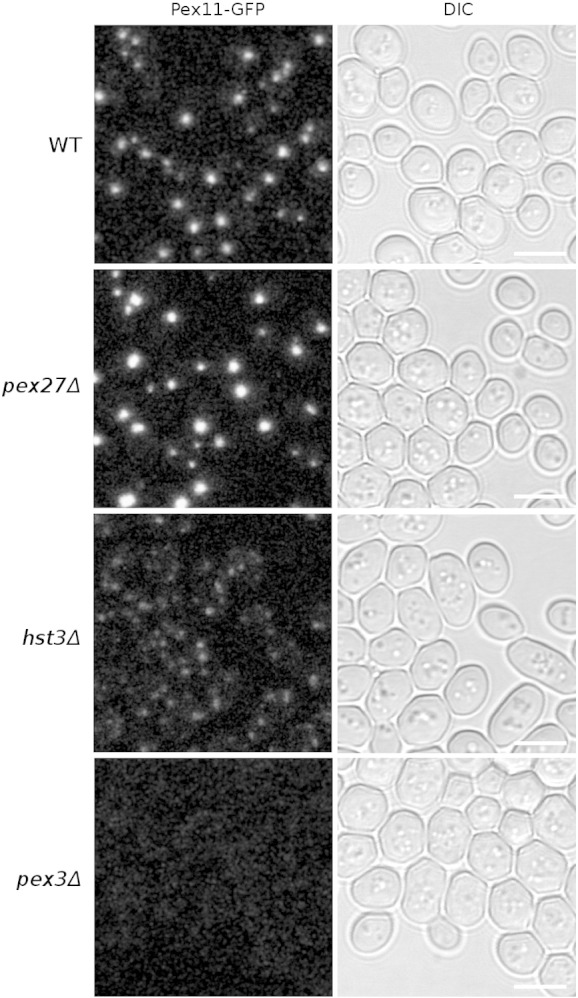
Pex11 localization patterns. Pex11 was genetically tagged with GFP in the library of 4292 non-essential gene deletion strains and 793 strains with temperature-sensitive alleles of 503 essential genes. In the majority of the strains, the localization pattern of Pex11-GFP was indistinguishable from the reference strain (WT), whereas in ~ 483 strains, it was significantly different. Three examples of different patterns are shown: *pex27*Δ with significantly more intense and larger peroxisomes, *hst3*Δ with more diffuse and less intense Pex11-GFP signal and *pex3*Δ with a much weaker signal. The same microscope settings were used to screen all the strains. Scale bars represent 5 μm.

The majority of tested mutants did not significantly affect Pex11-GFP localization compared to the reference strain, whereas abnormal Pex11-GFP localization patterns were assigned to the 10% (483) of strains with the phenotype farthest away from the wild type. Some examples of these are shown in Fig. 1. Further quantitative analysis revealed a group of 109 strains with the largest disruption of Pex11 localization (Supplementary Table 1). In the wild-type reference strain, typically one to a few peroxisomes per cell are present, and Pex11 is localized exclusively to these organelles. In cells lacking the *PEX27*/*YOR193W* gene, significantly larger peroxisomes were observed, and Pex11-GFP appears to be exclusively localized to the membranes of these peroxisomes as in the wild-type strain; however, the fluorescence intensity is markedly increased, indicating more condensed Pex11 protein localization. Pex27 is a peroxin known to regulate peroxisome size and number [17,18]; therefore, altered peroxisome morphology and consequently abnormal Pex11-GFP localization pattern are not surprising. On the other hand, in cells lacking the gene *HST3/YOR205W*, smaller and lower-intensity puncta of Pex11-GFP were observed. Hst3 is a protein from the sirtuin family and is involved in short-chain fatty acid metabolism [19]. This altered Pex11 localization in the *hst3*Δ mutant strain is intriguing, especially in the light of the known crosstalk between PPARs and sirtuins in humans [20].

The majority of tested mutants did not significantly affect Pex11-GFP localization compared to the reference strain, whereas abnormal Pex11-GFP localization patterns were assigned to the 10% (483) of strains with the phenotype farthest away from the wild type. Some examples of these are shown in Fig. 1. Further quantitative analysis revealed a group of 109 strains with the largest disruption of Pex11 localization (Supplementary Table 1). In the wild-type reference strain, typically one to a few peroxisomes per cell are present, and Pex11 is localized exclusively to these organelles. In cells lacking the *PEX27*/*YOR193W* gene, significantly larger peroxisomes were observed, and Pex11-GFP appears to be exclusively localized to the membranes of these peroxisomes as in the wild-type strain; however, the fluorescence intensity is markedly increased, indicating more condensed Pex11 protein localization. Pex27 is a peroxin known to regulate peroxisome size and number [17,18]; therefore, altered peroxisome morphology and consequently abnormal Pex11-GFP localization pattern are not surprising. On the other hand, in cells lacking the gene *HST3/YOR205W*, smaller and lower-intensity puncta of Pex11-GFP were observed. Hst3 is a protein from the sirtuin family and is involved in short-chain fatty acid metabolism [19]. This altered Pex11 localization in the *hst3*Δ mutant strain is intriguing, especially in the light of the known crosstalk between PPARs and sirtuins in humans [20].

**Fig. 2:**
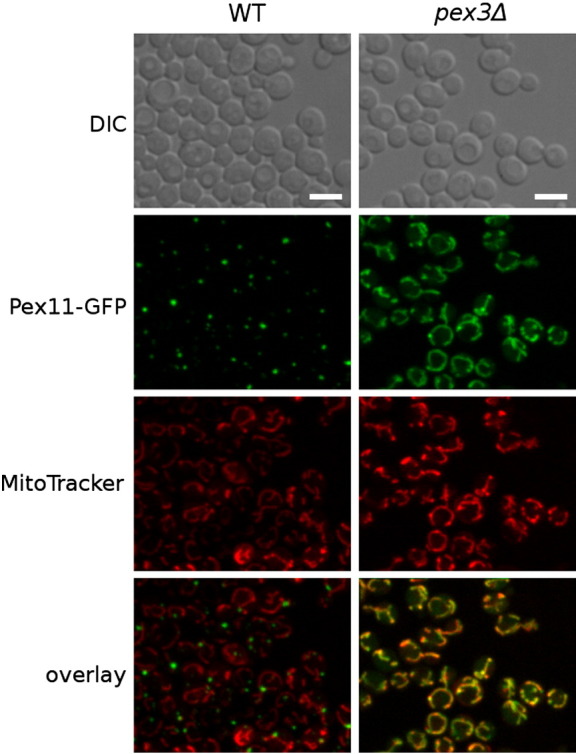
Pex11 is localized to mitochondria in *pex3*Δ cells lacking peroxisomes. Abnormal Pex11-GFP localization pattern was observed in *pex3*Δ cells in the screen (see Fig. 1). To determine the subcellular localization of Pex11 in *pex3*Δ strain, we used longer exposure times. Co-localization with a MitoTracker Red CMXRos mitochondrial marker largely overlaps with the localization of Pex11-GFP. Scale bars represent 5 μm.

Cells lacking Pex3 protein completely lack peroxisomes [21–23]. In our screen, only very low levels of the Pex11-GFP signal could be detected in cells lacking the *PEX3*/*YDR329C* gene with the microscope settings used for the genome-wide screen (Fig. 1). However, when we investigated this strain specifically, longer exposure times were used and a localization pattern of Pex11-GFP, resembling the shape of mitochondria, was observed (Fig. 2). Co-localization with a specific mitochondrial marker (MitoTracker Red CMXRos) confirmed a predominantly mitochondrial localization of Pex11-GFP in *pex3*Δ cells that lack peroxisomes. In the wild-type strain, no mitochondrial localization of Pex11-GFP was observed, indicating mis-localization of Pex11-GFP in the absence of the native target membrane.

### Computational analysis pipeline for microscopy images generated in the screen

**Supplementary Fig. 1:**
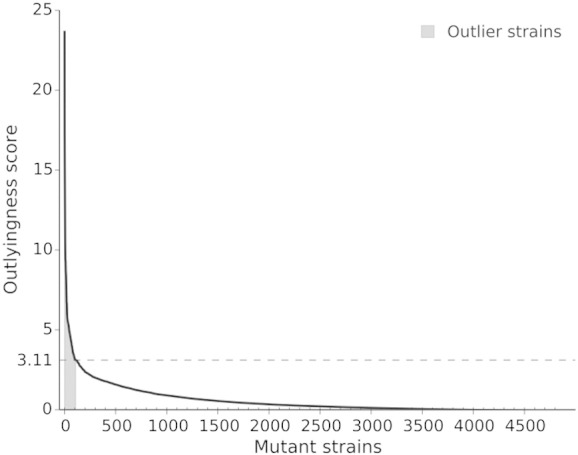
Ranking of the mutant strains according to their outlyingness score revealed a subgroup of 109 strains, corresponding to 104 genes, with most pronounced phenotypic changes relative to the reference strain. The cutoff value used in the outlier criterion was selected by searching for the first kink in the scores that were ordered by their magnitudes in descending order.

**Fig. 3:**
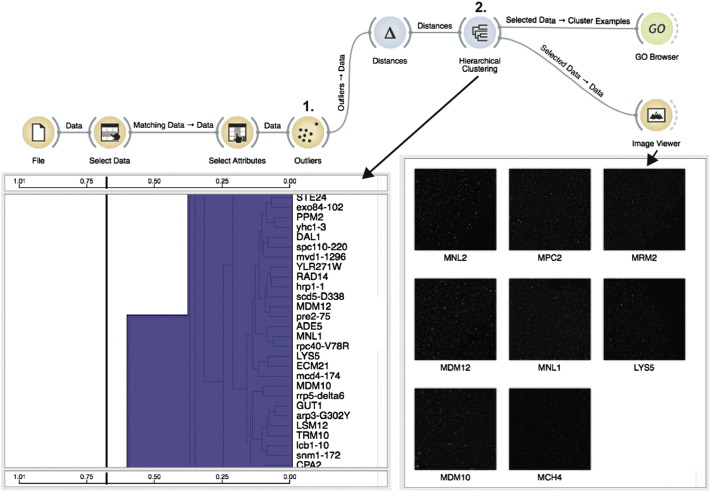
Conceptual workflow of genome-wide exploratory analysis of Pex11-GFP localization phenotype in Orange package. We defined a workflow consisting of several Orange widgets that collectively performed computational analysis of mutant strains generated in the screen. Outlier detection method (its corresponding Orange widget is marked with “1”) was applied to the preprocessed morphological profiles with the aim of identifying mutants with substantially different Pex11-GFP localization patterns from those of the reference strains. Hierarchical clustering (its corresponding Orange widget is marked with “2”) followed this step and clustered the genes according to the distance between their profiles. Images for one of the clusters are shown. Notably, the illustrated cluster contains strains for two genes, *MDM10* and *MDM12*, which encode two components of the ERMES complex. The clustering results were used for functional classification based on Gene Ontology of genes/proteins that affect the localization of Pex11.

**Fig. 4:**
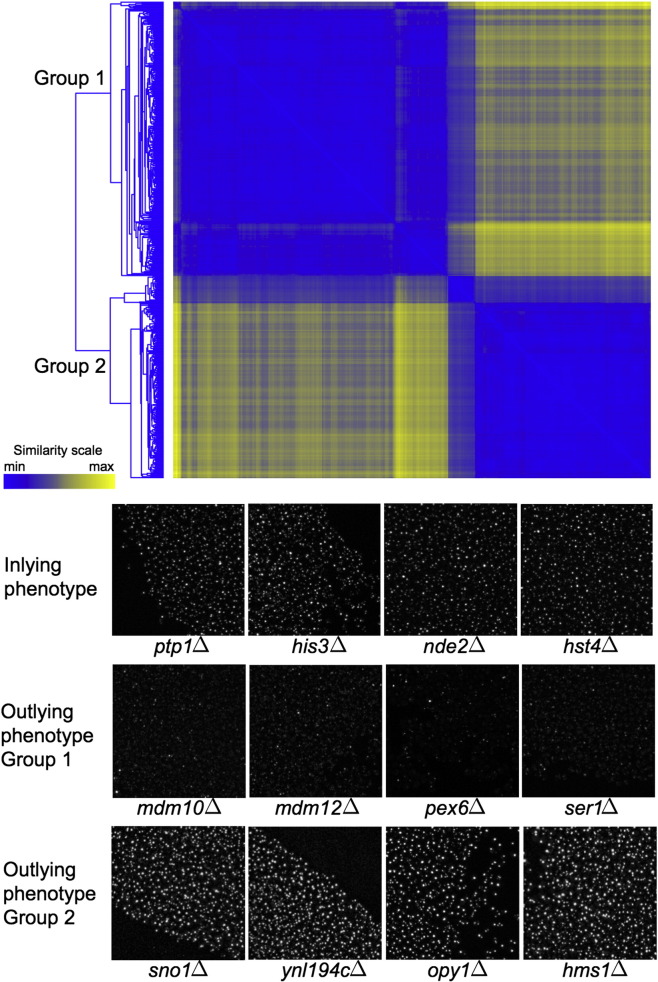
Partitioning of outlying mutant strains into groups based on morphological features extracted from their images. The organizational structure of outlying mutants found with hierarchical clustering revealed that there exist two groups of mutant strains with distinct Pex11 localization patterns at the highest level. One group of mutants exhibited Pex11-GFP localization patterns that were more diffused than the phenotypes of majority of the mutants (the inlying phenotype), whereas mutants from the second group had fewer but larger fluorescent objects that were of greater intensity compared to the inlying phenotype. Further statistical analysis that aimed to identify morphological features that discriminated the identified groups successfully pinpointed relevant features. Representative members of the two groups are shown along with representative mutants with inlying phenotype.

The computational analysis pipeline for images generated in the genome-wide screen involved image preprocessing and analysis, data mining for outlier detection and clustering, data visualization and gene set enrichment analysis (Fig. 3). The pipeline designed in Orange data mining toolbox [24] allowed us to systematically analyze and explore data and to define a workflow that could be used in subsequent studies. This result advances the recently proposed analysis protocol [25] that is based on individual inspection of strain images and their visual comparison with reference strains by unbiased observers. In our approach, the phenotypes were quantified with vectors containing morphological features extracted from high-content microscopy images. We then applied an outlier detection algorithm to identify mutant strains with Pex11-GFP localization phenotypes that were substantially different from phenotypes observed in majority of the mutants. To rank mutant strains according to the degree of altered Pex11-GFP localization effects, we defined the outlyingness score (see Materials and Methods) and evaluated it for every strain from the collection of 483 strains with abnormal Pex11 localization patterns (Supplementary Table 1). This analysis revealed a subgroup of 109 strains, corresponding to 104 genes, with most pronounced phenotypic changes relative to the reference strain (Supplementary Fig. 1). We assessed the quality of our outlier detection approach by estimating the proportion of reference strains that the algorithm incorrectly identified as outliers. There were a total of 6 reference strains, out of 211 reference strains, that ranked among the top 10% strains with altered localization patterns. Although this result is an optimistic quality estimate, it suggests a rather low 1% pollution of the ranked phenotype list. Hence, we can conclude that the proposed outlier detection approach performs well. We also compared the outlyingness scores of reference strains with the distribution of scores of mutant strains and concluded that they were significantly different (*p* = 0.006). Strains with outlying phenotypes were partitioned into two groups that were revealed by hierarchical clustering (Fig. 4).

The computational analysis pipeline for images generated in the genome-wide screen involved image preprocessing and analysis, data mining for outlier detection and clustering, data visualization and gene set enrichment analysis (Fig. 3). The pipeline designed in Orange data mining toolbox [24] allowed us to systematically analyze and explore data and to define a workflow that could be used in subsequent studies. This result advances the recently proposed analysis protocol [25] that is based on individual inspection of strain images and their visual comparison with reference strains by unbiased observers. In our approach, the phenotypes were quantified with vectors containing morphological features extracted from high-content microscopy images. We then applied an outlier detection algorithm to identify mutant strains with Pex11-GFP localization phenotypes that were substantially different from phenotypes observed in majority of the mutants. To rank mutant strains according to the degree of altered Pex11-GFP localization effects, we defined the outlyingness score (see Materials and Methods) and evaluated it for every strain from the collection of 483 strains with abnormal Pex11 localization patterns (Supplementary Table 1). This analysis revealed a subgroup of 109 strains, corresponding to 104 genes, with most pronounced phenotypic changes relative to the reference strain (Supplementary Fig. 1). We assessed the quality of our outlier detection approach by estimating the proportion of reference strains that the algorithm incorrectly identified as outliers. There were a total of 6 reference strains, out of 211 reference strains, that ranked among the top 10% strains with altered localization patterns. Although this result is an optimistic quality estimate, it suggests a rather low 1% pollution of the ranked phenotype list. Hence, we can conclude that the proposed outlier detection approach performs well. We also compared the outlyingness scores of reference strains with the distribution of scores of mutant strains and concluded that they were significantly different (*p* = 0.006). Strains with outlying phenotypes were partitioned into two groups that were revealed by hierarchical clustering (Fig. 4).

**Fig. 5:**
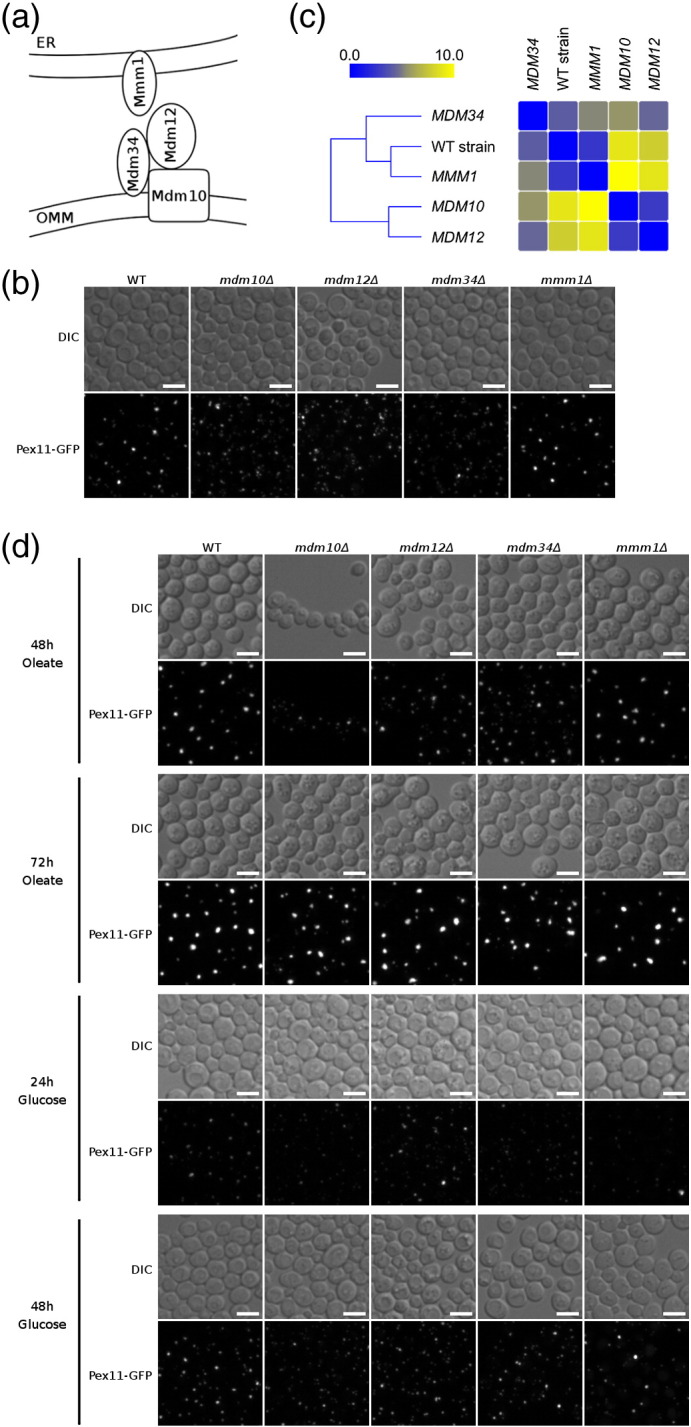
Mitochondrial/cytosolic components of the ERMES complex influence subcellular localization of Pex11. (a) Scheme depicting localization of ERMES complex components. Mdm10 and Mdm34 are outer mitochondrial membrane proteins, Mdm12 is a cytosolic component and Mmm1 is integral to the ER membrane. (b) Localization of Pex11 in glucose-grown cells in ERMES complex components deletion mutants. While Pex11-GFP localization pattern in *mmm1*Δ is not significantly different from the reference strain (WT), cells of the other three mutants contain a number of fainter and diffuse puncta. Localization patterns of Pex11-GFP in the *mdm10*Δ, *mdm12*Δ and *mdm34*Δ strains are similar to each other and, at the same time, significantly different from the reference strain. Notably, the localization patterns of Pex11-GFP in *mdm10*Δ and *mdm12*Δ strains seem almost identical. The total amount of Pex11-GFP signal, however, does not seem to be significantly altered in the ERMES mutant strains. (c) Heat-map table showing pairwise distances of the Pex11-GFP localization patterns, quantified using CellProfiler-derived vectors containing morphological features, between the *mdm10*Δ, *mdm12*Δ, *mdm34*Δ and *mmm1*Δ strains and a reference strain. (d) Localization of Pex11 under conditions of peroxisome proliferation and pexophagy in the ERMES complex components deletion mutants. Cells exposed to oleate induce peroxisome proliferation. Under these conditions, the localization pattern of Pex11-GFP becomes indistinguishable between all four ERMES complex components deletion mutants and the reference strain (WT). When cells are transferred back into glucose-containing medium, peroxisomes are degraded in the process of pexophagy. Under these conditions, the localization pattern in *mdm10*Δ, *mdm12*Δ and *mdm34*Δ strains again becomes significantly different, with more Pex11-GFP patches with weaker signal compared to the reference strain. Scale bars represent 5 μm.

The first group of strains (Fig. 4) displayed a more diffused Pex11-GFP localization pattern. Four subgroups were identified using the second round of hierarchical clustering within this group, and one of the subgroups of mutants with a similar Pex11-GFP localization phenotype, consisting of 56 genes, was significantly enriched (32 genes; *p* = 0.011)¶ for genes encoding proteins with nuclear localization, of which one-half are involved in mRNA splicing (*CEF1*, *CWC21*, *DBR1*, *PRP19*, *YHC1*), transcription from RNA polymerase II promoter (*HDA1*, *IXR1*, *NRM1*, *RPT3*, *TAF5*) or intracellular protein degradation (*APC11*, *PUP1*, *RPN13*, *RPT3*, *SEC13*). Another subgroup of 59 mutants with a specific Pex11-GFP localization pattern was most significantly enriched for genes encoding components of the ERMES complex, *MDM10* and *MDM12*. The ERMES complex consists of four structural components: Mdm10 and Mdm34 bound to the mitochondrial outer membrane, cytosolic Mdm12 and Mmm1 bound to the endoplasmic reticulum (ER) [26] (Fig. 5a). Deletions of *MDM34* and *MMM1* were not identified by the computational analysis, but visual inspection revealed that Pex11-GFP localization is also more diffuse in the *mdm34*Δ strain compared to wild-type cells (see also below). Notably, *YGL218W*, a dubious open reading frame of which 93% overlaps with the *MDM34* gene, was identified in the screen.

The second group of mutants exhibited less apparent localization patterns and was subject to subsequent computational analysis to find cluster-specific morphological features that distinguished this group from the reference. This analysis revealed the importance of features reporting the intensity of localization patterns: Pex11-GFP localization spots in the strains belonging to the second group were sparser but more intense (Fig. 4). This group contained the abovementioned *pex27*Δ strain, as well as another strain with deleted peroxin-encoding gene, *inp1*Δ. The most significantly enriched functional group of genes within this second group, however, comprises genes encoding components of the Rpd3L histone deacetylase complex, namely, *PHO23*, *DEP1*, *RXT2* and *RXT3*.

### Pex11-GFP localization and peroxisome morphology in the ERMES complex mutants

**Supplementary Fig. 2:**
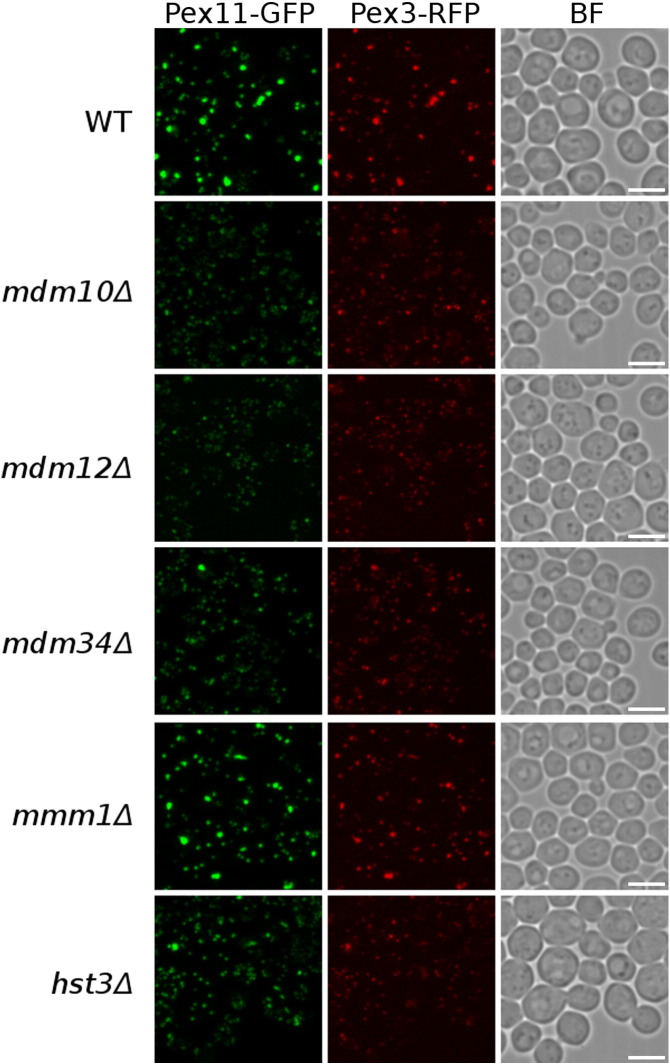
Localization of Pex11-GFP and Pex3-RFP in mutants of ERMES complex components. Three ERMES complex components mutants (*mdm10*Δ, *mdm12*Δ and *mdm34*Δ) have aberrant peroxisome morphology compared to the wild-type strain. Deletion of *MMM1*, on the other hand, does not cause a significant difference in the phenotype. The phenotype observed in the *hst3*Δ strain clustered to the same outlier group as the ERMES complex mutants and similarly leads to a general defect in peroxisome morphology. Scale bars represent 5 μm.

We next investigated in more detail the Pex11/peroxisome phenotype of mutants lacking components of the ERMES complex, since this complex has been previously implicated in the interaction of peroxisomes with other organelles [27]. Deletion strains *mdm10*Δ, *mdm12*Δ, *mdm34*Δ and *mmm1*Δ, all harboring the *PEX11-GFP* cassette, were imaged under different growth conditions. When grown in glucose-containing medium, deletion of the mitochondrial and cytosolic ERMES complex components Mdm10, Mdm12 and Mdm34 (Fig. 5a) caused a significantly different Pex11-GFP localization pattern from the one observed in wild-type cells (Fig. 5b): in addition to the large puncta with an intense signal seen also in the reference strain, numerous additional but weaker puncta were observed. Pex11-GFP localization in *mdm10*Δ and *mdm12*Δ strains was very similar, which is in contrast to the *mdm34*Δ mutant that showed less focal highly intense Pex11-GFP signal puncta. Notably, absence of the ER component of the ERMES complex, Mmm1, did not affect the Pex11-GFP localization pattern at all (Fig. 5b). We calculated the pairwise distances of the Pex11-GFP localization patterns that were quantified using CellProfiler-derived vectors describing morphological features in the *mdm10*Δ, *mdm12*Δ, *mdm34*Δ, *mmm1*Δ and wild-type strains (Fig. 5c). The calculated distances confirmed the interpretation of the visual inspection of the images described above: the Pex11-GFP localization patterns in the *mdm10*Δ and *mdm12*Δ are extremely similar but are very different from the wild-type strain, to which the *mmm1*Δ strain is very similar. The pattern in the *mdm34*Δ strain is different from those in the other four strains. The observed differences between the ERMES complex mutants and the wild-type strain first seemed specific for the Pex11-GFP localization, as they were not identified as significant in our previous study with a luminal peroxisomal marker GFP-PTS1 [16]. However, when another general peroxisomal marker, Pex3-RFP, was used, it became clear that the three ERMES complex mutants have different peroxisome morphology (Supplementary Fig. 2) in agreement with a recent study [28].

We next investigated in more detail the Pex11/peroxisome phenotype of mutants lacking components of the ERMES complex, since this complex has been previously implicated in the interaction of peroxisomes with other organelles [27]. Deletion strains *mdm10*Δ, *mdm12*Δ, *mdm34*Δ and *mmm1*Δ, all harboring the *PEX11-GFP* cassette, were imaged under different growth conditions. When grown in glucose-containing medium, deletion of the mitochondrial and cytosolic ERMES complex components Mdm10, Mdm12 and Mdm34 (Fig. 5a) caused a significantly different Pex11-GFP localization pattern from the one observed in wild-type cells (Fig. 5b): in addition to the large puncta with an intense signal seen also in the reference strain, numerous additional but weaker puncta were observed. Pex11-GFP localization in *mdm10*Δ and *mdm12*Δ strains was very similar, which is in contrast to the *mdm34*Δ mutant that showed less focal highly intense Pex11-GFP signal puncta. Notably, absence of the ER component of the ERMES complex, Mmm1, did not affect the Pex11-GFP localization pattern at all (Fig. 5b). We calculated the pairwise distances of the Pex11-GFP localization patterns that were quantified using CellProfiler-derived vectors describing morphological features in the *mdm10*Δ, *mdm12*Δ, *mdm34*Δ, *mmm1*Δ and wild-type strains (Fig. 5c). The calculated distances confirmed the interpretation of the visual inspection of the images described above: the Pex11-GFP localization patterns in the *mdm10*Δ and *mdm12*Δ are extremely similar but are very different from the wild-type strain, to which the *mmm1*Δ strain is very similar. The pattern in the *mdm34*Δ strain is different from those in the other four strains. The observed differences between the ERMES complex mutants and the wild-type strain first seemed specific for the Pex11-GFP localization, as they were not identified as significant in our previous study with a luminal peroxisomal marker GFP-PTS1 [16]. However, when another general peroxisomal marker, Pex3-RFP, was used, it became clear that the three ERMES complex mutants have different peroxisome morphology (Supplementary Fig. 2) in agreement with a recent study [28].

We next investigated the Pex11-GFP localization pattern under conditions of peroxisome proliferation (Fig. 5d) under which Pex11 has a well-defined function. While the function of rather highly expressed Pex11 is currently unclear in cells growing on glucose, it is absolutely required for tubulation of peroxisomal membranes upon induction with oleic acid [9,10]. Notably, when cells were exposed to oleic acid to induce peroxisome proliferation, peroxisome morphology and Pex11-GFP localization patterns did not differ between the wild-type strain and the ERMES complex mutants after 72 h. A weak effect of the mutants was still observed after 48 h, but this was most likely a remnant of the glucose growth phase as peroxisomes are known to respond slowly to addition of oleic acid and are not being degraded under such conditions so that the glucose-related phenotype can still be observed. When cells were shifted back to glucose-containing medium to induce pexophagy, the specific Pex11-GFP localization pattern re-emerged after 48 h in the *mdm10*Δ, *mdm12*Δ and *mdm34*Δ mutants. This clearly demonstrates that the presence of the ERMES complex is only important for Pex11 localization in cells grown on glucose, and this indicates a different role for Pex11 in glucose-grown cells, which is linked to the ERMES complex/mitochondria.

**Supplementary Fig. 3:**
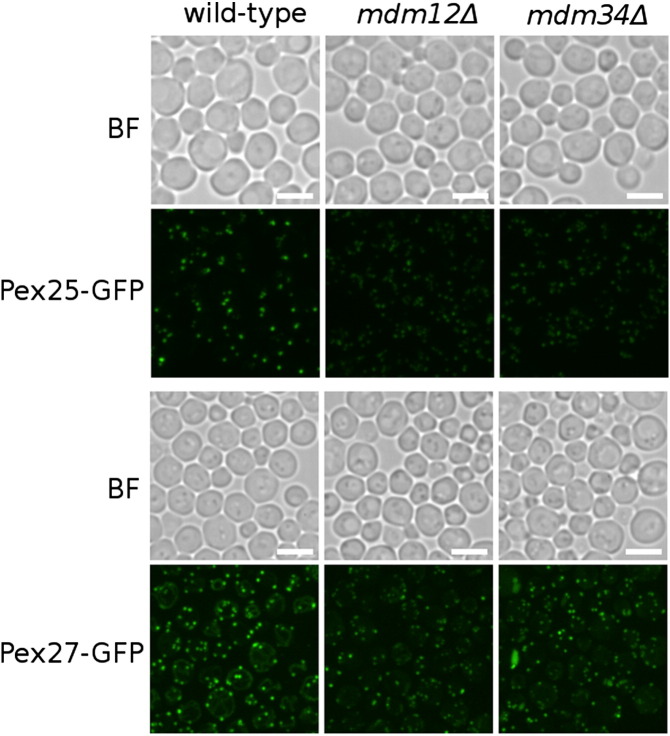
Mutations of the ERMES complex components affect the localization of Pex25-GFP and Pex27-GFP. The localization pattern on Pex25-GFP in *mdm12*Δ and *mdm34*Δ cells is comparable to the mutants' effects on Pex11-GFP (see Fig. 5b and Supplementary Fig. 2). The localization pattern of Pex27-GFP in these two mutants differs from that observed for Pex25-GFP and Pex11-GFP but is at the same time different from the wild-type pattern. Scale bars represent 5 μm. Mutations of the ERMES complex components affect the localization of Pex25-GFP and Pex27-GFP. The localization pattern on Pex25-GFP in *mdm12*Δ and *mdm34*Δ cells is comparable to the mutants' effects on Pex11-GFP (see Fig. 5b and Supplementary Fig. 2). The localization pattern of Pex27-GFP in these two mutants differs from that observed for Pex25-GFP and Pex11-GFP but is at the same time different from the wild-type pattern. Scale bars represent 5 μm.

Pex25 is structurally similar to Pex11 and has largely overlapping cellular functions [17], whereas the paralog of Pex25, Pex27, has a distinct cellular role [18]. We therefore asked the question whether their localization patterns are similar to the one of Pex11 in the ERMES mutants. Pex25-GFP and Pex27-GFP markers were imaged in the *mdm12*Δ and *mdm34*Δ strains, and the pattern of Pex25-GFP was essentially the same as that of Pex11-GFP, whereas the pattern of Pex27-GFP was different, less diffuse, yet also different from the wild-type phenotype (Supplementary Fig. 3).

Pex25 is structurally similar to Pex11 and has largely overlapping cellular functions [17], whereas the paralog of Pex25, Pex27, has a distinct cellular role [18]. We therefore asked the question whether their localization patterns are similar to the one of Pex11 in the ERMES mutants. Pex25-GFP and Pex27-GFP markers were imaged in the *mdm12*Δ and *mdm34*Δ strains, and the pattern of Pex25-GFP was essentially the same as that of Pex11-GFP, whereas the pattern of Pex27-GFP was different, less diffuse, yet also different from the wild-type phenotype (Supplementary Fig. 3).

### Expression of *PEX11* is not affected by ERMES complex mutations

**Supplementary Fig. 4:**
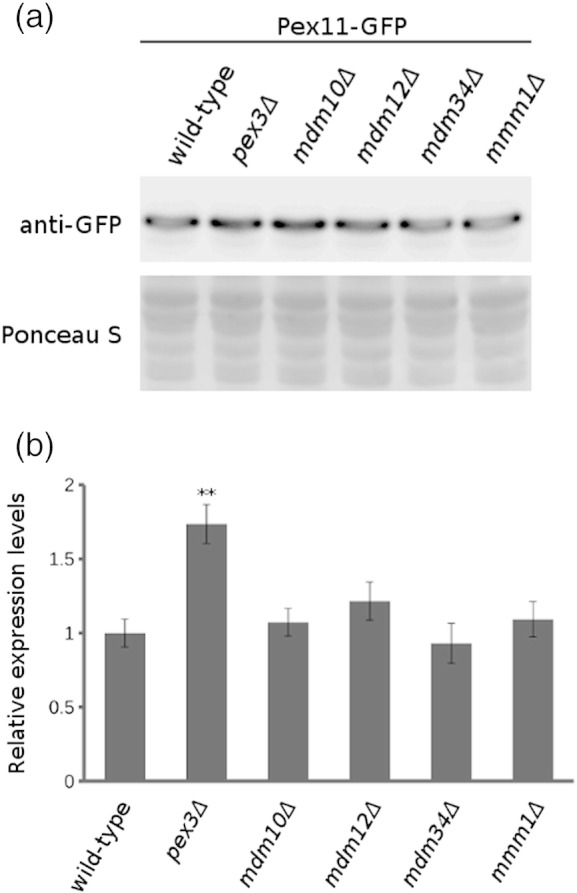
ERMES complex mutants do not affect Pex11 protein and *PEX11* gene expression levels. (a) Pex11 protein levels in the wild-type and *pex3*Δ*, mdm10*Δ*, mdm12*Δ*, mdm34*Δ and *mmm1*Δ strains. The levels of Pex11-GFP were assessed by Western blotting using anti-GFP antibody. Ponceau S staining of the membrane was used as a loading control. (b) Relative expression levels of *PEX11* mRNA in the wild-type strain and in the *pex3*Δ*, mdm10*Δ*, mdm12*Δ*, mdm34*Δ and *mmm1*Δ deletion mutants. The values were normalized to the wild-type expression levels, and the error bars represent standard deviations from mean values. **p < 0.01.

We hypothesized that altered gene expression could cause the mis-localization of Pex11 in the mutant strains. Therefore, to better understand the mechanism for the altered Pex11-GFP localization, we first determined the level of Pex11 protein in strains mutated in genes encoding ERMES complex components by Western blot. Protein levels were unaffected in all the tested strains. Additionally, the level of *PEX11* gene expression was determined by quantitative real-time PCR in the same strains and, again, no differences were observed (Supplementary Fig. 4).

We hypothesized that altered gene expression could cause the mis-localization of Pex11 in the mutant strains. Therefore, to better understand the mechanism for the altered Pex11-GFP localization, we first determined the level of Pex11 protein in strains mutated in genes encoding ERMES complex components by Western blot. Protein levels were unaffected in all the tested strains. Additionally, the level of *PEX11* gene expression was determined by quantitative real-time PCR in the same strains and, again, no differences were observed (Supplementary Fig. 4).

No significant difference in Pex11 protein and *PEX11* gene expression levels in the ERMES complex mutants is in agreement with the estimated protein abundance from the microscopy data (Fig. 5b). Thus, changes in Pex11-GFP localization are not due to altered levels of gene expression in the mutants. Notably, approximately 70% increased expression of *PEX11* gene was detected in the *pex3*Δ strain, which was used for comparison, whereas the Pex11 protein abundance in this strain remained the same. The increased gene expression thus indicates reduced translation efficiency or protein stability in the absence of a peroxisomal membrane.

### Pex11 and Mdm34 physically interact

**Fig. 6:**
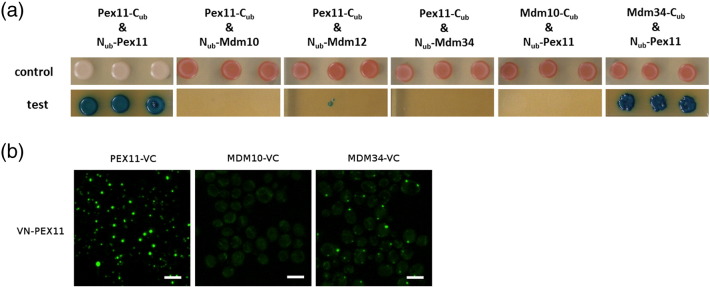
Pex11 and Mdm34 physically interact. (a) MYTH method was used to test the physical interactions of Pex11 with the ERMES complex components. Bait proteins were tagged with the C_ub_ C-terminally, and prey proteins were tagged with N_ub_ N-terminally. Homodimerization of Pex11 was used as a positive control. Growth of the strain with Mdm34 as the bait protein and Pex11 as the prey protein revealed that Pex11 and Mdm34 physically interact. Three independent experiments were performed and the same result was obtained in all of them; a representative result is shown in the figure. Control: SD − Leu − Trp. Test: SD − Leu − Trp − Ade − His + 5-bromo-4-chloro-3-indolyl-β,d-galactopyranoside. (b) The physical interaction between Pex11 and Mdm34 was confirmed with the BiFC assay. Pex11 was tagged at its N-terminus with the N-terminal part of Venus (VN) and assayed against Pex11 (positive control), and Mdm10 and Mdm34 were tagged at their C-termini with the C-terminal part of Venus (VC). As with MYTH, an interaction was observed between Pex11 and Mdm34. Scale bars represent 5 μm.

We next reasoned that there may be a direct physical interaction between the mitochondrial and/or cytosolic components of the ERMES complex and Pex11, which, when disrupted by the deletion of one of the complex components, could result in Pex11 mis-localization. Membrane yeast two-hybrid (MYTH) analysis [29–32] was used to systematically test the physical interaction of Pex11 with the components of the ERMES complex. In this analysis, Pex11 was used as the bait protein and tagged with C_ub_ at the C-terminus to test its interaction with N-terminally N_ub_-tagged Mdm10, Mdm12 and Mdm34. As a positive control, Pex11 was used as the prey protein (*PEX11*-C_ub_withN_ub_-*PEX11*) as it is known to homodimerize [33], which can be readily observed using MYTH (U.P., unpublished results). In addition, Mdm10 and Mdm34 were used as the baits and analyzed for the interaction with Pex11. Of all the tested combinations, growth of the colonies co-expressing Pex11-C_ub_ and N_ub_-Pex11 and Mdm34-C_ub_ and N_ub_-Pex11 was repeatedly observed (Fig. 6a). To test the observed interaction with an independent method, we used bimolecular fluorescence complementation (BiFC) assay [34] and we assayed Pex11 against Mdm34, Mdm10 and Pex11 itself as a positive control. In accordance with MYTH results, interaction between Pex11 and Mdm34 was observed with BiFC, whereas Pex11 and Mdm10 did not interact with each other (Fig. 6b). These experiments thus clearly show that Pex11 can directly physically interact with Mdm34. The results of this experiment also indicate that the N-terminal part of Mdm34 either is not exposed to the cytosol or has to be intact in order to interact with Pex11, as no interaction was observed with N-terminally tagged Mdm34.

### Pex11 is involved in establishing the peroxisome/mitochondria contact sites

**Fig. 7:**
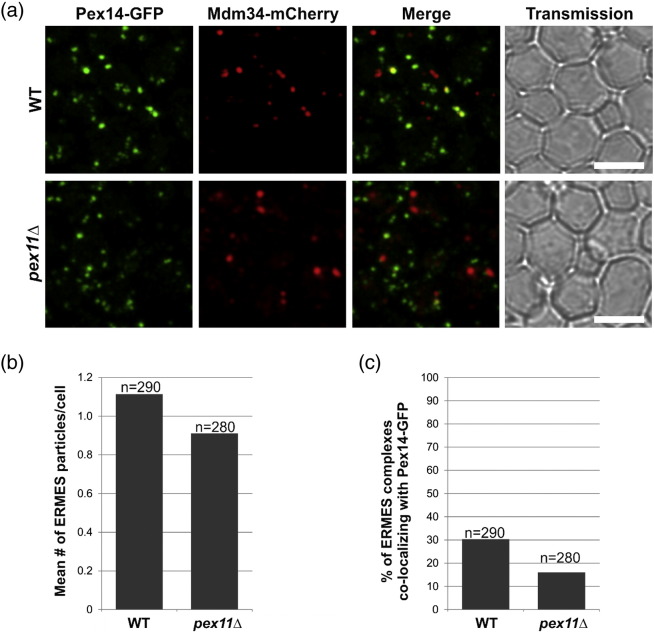
Pex11 is involved in establishing the peroxisome/mitochondria contact sites. (a) The apparent co-localization events between peroxisomes (Pex14-GFP as marker) and mitochondria/ERMES complex (Mdm34-mCherry as marker) were quantified in the wild-type and *pex11*Δ strains. (b) The mean number of ERMES particles per cell did not differ significantly between the two strains. (c) The percentage of Pex14-GFP marked peroxisomes apparently co-localizing with the ERMES foci is reduced from 30% in wild-type cells to 15% in *pex11*Δ cells. Scale bars represent 5 μm.

Cohen *et al*. showed that approximately one-third of peroxisomes were adjacent to ERMES foci [28]. To test whether Pex11 is involved in establishing such contact sites, presumably through interaction with Mdm34, we counted the apparent co-localization events between Pex14-GFP as a peroxisomal marker and Mdm34-mCherry as the ERMES marker in the wild-type and *pex11*Δ strains (Fig. 7). On average, 0.9 ERMES foci per cell were observed in the wild-type strain, and 1.1 foci were observed in *pex11*Δ cells (Fig. 7b). This difference is not statistically significant. The portion of Pex14-GFP marked peroxisomes apparently co-localizing with the ERMES foci was then determined and was found to be 30% in the wild-type cells, in agreement with the previous report [28]. In *pex11*Δ cells, however, this portion was significantly lower as only 15% of the peroxisomes apparently co-localized with the ERMES foci (Fig. 7c). We can therefore conclude that Pex11 plays a role in establishing the contact sites between peroxisomes and mitochondria through the ERMES complex.

## Discussion

A genome-wide high-content microscopy study was performed to elucidate the molecular function of Pex11 protein. The results of this study provided new knowledge on Pex11 and suggested a series of focused follow-up experiments to elucidate the observed functional interaction of Pex11 with components of the ERMES complex in greater detail. Since from the screen alone we could not directly discriminate whether the identified mutants cause a general peroxisomal or a Pex11-specific defect, additional protein markers of peroxisomes were used in the follow-up experiments showing that, for the most part, changes in Pex11-GFP localization pattern were a reflection of changes in peroxisome morphology.

Pex11-GFP was associated with intracellular membrane structures in essentially all analyzed mutant strains, with significant deviations among the mutants regarding signal intensity and subcellular morphology. This is inline with recent findings that human PEX11γ protein is a transmembrane protein with two transmembrane domains [15] and with our own MYTH study on yeast Pex11 that also indicated two transmembrane domains (U.P., unpublished results); interestingly, computational prediction of Pex11 transmembrane domains is inconclusive. Notably, none of the localization patterns corresponded to a typical ER or nuclear envelope morphology, which could have been anticipated given the proposed evolutionary conservation between yeast Pex11 and mammalian nuclear receptors. In the *pex3*Δ cells, which lack peroxisomes, Pex11 is localized to mitochondria. This result indicates that Pex11 has some affinity to mitochondria, potentially through known interactions with the mitochondrial proteins, Fis1 and Dnm1 [9,10], and with Tom22 [35]. Notably, the interaction of Pex11 with Tom22 was found to be stronger in the absence of peroxisomal membranes, such as in the *pex3*Δ mutant [35]. Somewhat increased *PEX11* gene expression was observed in *pex3*Δ cells compared to the wild type, but on the other hand, the Pex11 protein abundance in this strain was significantly lower than that in the wild type. We propose that this phenomenon occurs because of a feedback mechanism activating *PEX11* gene expression when Pex11 is not inserted into the peroxisomal membrane in sufficient quantity and because of a simultaneous increase in Pex11 protein degradation that is significantly increased when peroxisomes are not present in yeast cells [36].

The ERMES complex is thought to provide a tether and to facilitate the exchange of molecules between the ER and mitochondria [26]. Here, we show that Mdm34, a mitochondrial component of the ERMES complex, can bind directly to Pex11. This interaction was observed with MYTH and BiFC methods, and in all tested cases, Mdm34 had to be tagged on the C-terminal side, as the interaction was never observed with the N-terminally tagged Mdm34. This indicates that a non-modified N-terminus of Mdm34 is required for the interaction and that the interaction between Mdm34 and Pex11 occurs most likely close to the N-terminal part of Mdm34. The Pex11 localization results demonstrate that the mitochondrial and cytosolic components (Mdm10, Mdm34 and Mdm12), but not the ER component (Mmm1), of the ERMES complex affect the localization pattern of Pex11. Gem1 protein has also been identified as an ERMES complex component and has been proposed as its regulatory subunit [37]. Our study, however, did not unveil any differences of the Pex11-GFP localization pattern between *gem1*Δ and the wild-type strain, indicating that Gem1 likely has no direct function in the ERMES-mediated peroxisome–mitochondria connection. We propose that cytosolic and mitochondrial components of the ERMES complex play a role in linking mitochondria to peroxisomes via Pex11 in a process, which is independent of Mmm1 and Gem1; alternatively, absence of Mmm1 (and Gem1) does not disrupt the ERMES complex in a way that prevents Pex11 binding and localization. Nevertheless, the results obtained in this study point to a specific peroxisome–mitochondria contact that involves components of the ERMES complex, also in agreement with recent findings [28]. Notably, this study showed that Pex11 has an important role in establishing the contacts between peroxisomes and mitochondria as the number of observed apparent contacts between these organelles is significantly reduced in the absence of Pex11. The reason that the apparent contacts are still present in the *pex11*Δ strain could be partly due to the buffering by another peroxisomal protein, for example, Pex25.

Quantitative analysis of the image data by the computational analysis pipeline offers an insight at a level deeper from mere visual genotype–phenotype correlations. The quantitative analysis (Fig. 5c) confirmed that the Pex11-GFP localization patterns in *mdm10*Δ and *mdm12*Δ strains are virtually identical, whereas the pattern in the *mdm34*Δ is significantly different. These results support a model by which physical interaction between Mdm34 and Pex11 forms a peroxisome–mitochondria tether: in the absence of Mdm34, Pex11 has no binding partner on the mitochondrial membrane and peroxisomes are localized more diffusely. In the absence of Mdm12 or Mdm10, Pex11 can bind to Mdm34, but since the ERMES complex is not complete, some of the peroxisomes/Pex11 protein molecules are still mis-localized. Notably, this only occurs in cells growing in the presence of glucose, but this is not observed when peroxisome proliferation is induced by the addition of fatty acids to the medium, in the absence of glucose. Thus, in yeast cells growing in glucose media, peroxisome morphology and Pex11 localization is determined by the Pex11–Mdm34/ERMES interaction. This interaction apparently does not influence the recruitment of the membrane fission machinery that is recruited in a Pex11-dependent manner in the presence of fatty acids [9,10], as ERMES complex mutants did not cause a peroxisome morphology phenotype in the oleic acid induction experiment. It was recently shown that peroxisomes are juxtaposed with mitochondria and that the ERMES complex is involved in this interorganelle connection [28]. Our results strongly suggest that Pex11 and Mdm34 form the tether for this juxtaposition. Given the structural resemblance and proposed homology to the PPAR ligand binding domain, Pex11 could be a sensor of the metabolic state of the peroxisome matrix. Our unpublished results show that the intracellular concentration of acetyl-coenzyme A is affected by Pex11 activity in cells growing on glucose, and acetyl-coenzyme A is thus a possible signaling molecule in this mechanism, in agreement with a previously proposed function of Pex11 as a transporter of a signaling molecule with a source in fatty acid β-oxidation that modulates the number of peroxisomal structures in a cell [38]. Following the shift into medium with oleate, different signals predominate in the guidance of the localization of Pex11, which is then required for tubulation and fission of the peroxisomal membrane. *PEX11* gene expression and protein abundance are also significantly higher in cells growing in oleate-containing media. The results of the present study, in combination with thoroughly described roles of Pex11 in oleate-grown cells, demonstrate a new and different role of Pex11 in yeast cells growing on glucose.

A link between mitochondria and peroxisomes has recently been identified in the study of autophagy (pexophagy), which requires Pex11-dependent peroxisomal division that occurs on sites of mitochondrion–peroxisome interactions [39,40]. In humans, mitochondrial myopathy has been linked to the peroxisomal disorder, Zellweger syndrome [41], supporting the importance of the findings of this study also from the biomedical perspective.

## Materials and Methods

### Yeast strains, plasmids and growth conditions

The yeast *S. cerevisiae* haploid deletion collection (YKO; *MATa xxx*∷*KanMX his3Δ1 leu2Δ0 met15Δ0 ura3Δ0*) [42,43] and the collection of temperature-sensitive essential gene mutants (TS; *MATa xxx-ts*∷*KanMX his3Δ1 leu2Δ0 met15Δ0 ura3Δ0*) [44] were grown on YPD medium (1% yeast extract, 2% peptone, 2% glucose and 2% agar) containing 200 mg/L G418 (Formedium, UK) at 30 °C and 25 °C, respectively, unless stated otherwise. Both collections were a gift from Dr. Charlie Boone (University of Toronto, Canada). Individual deletion strains used in the study were taken from the YKO collection. Double mutants obtained through synthetic genetic array analysis were grown on YPD medium containing 200 mg/L G418 and 100 mg/L clonNAT (Werner BioAgents, Germany).

The strain BY4741 and plasmids pFA6a-KANMX6-P_RPL7B_-VN and pFA6a-VC-HIS3MX6 were used for the BiFC assay [34,45].

Pex14-GFP strain [46] and plasmid pFa6a-link-mCherry-CaURA3 (kind gift from Dr. Brenda Andrews) were used to test the peroxisome/mitochondria contact sites. For additional microscopy experiments, strains Pex3-RFP, Pex25-GFP and Pex27-GFP were used [46].

For the MYTH assay, yeast strain NMY51 and plasmids pAMBV4 and pPR3-N were used [31].

For peroxisome proliferation induction, minimal media containing 3% glycerol, 0.1% oleate and 1% Brij 35 was used.

### Strain and plasmid construction

All oligonucleotide primers used for strain construction are listed in Supplementary Table 2.

All oligonucleotide primers used for strain construction are listed in Supplementary Table 2.

For high-content microscopy, mGFP, together with the NatMX nourseothricin resistance cassette, was PCR amplified from pFA6a-mGFP-NATMX plasmid in order to create a mutant strain collection harboring *PEX11* C-terminally tagged with the monomeric GFP [47] and transformed into the AJY217 parental strain (MATα *can1Δ*∷*MFA1pr-HIS3 lyp1Δ his3Δ1 leu2Δ0 ura3Δ0 met15Δ0*). Primers pex11_gfp_nat_UP and pex11_gfp_nat_DN used for mGFP selection marker cassette amplification contain homology regions to the 3′ end of the *PEX11* open reading frame to allow in-frame fusion of the GFP tag. Correct insertion by homologous recombination was verified by PCR amplification of the insert's flanking regions using primer pairs pex11_gfp_nat_PR1/pex11_gfp_nat_PR2 and pex11_gfp_nat_PR3/pex11_gfp_nat_PR4. The obtained query strain was crossed to the YKO and TS collections, and double mutants were generated following the standard synthetic genetic array protocol described in Tong *et al.* [48]. Plate pinning was performed using a pinning robot (Adept Plus, Slovenia) with floating pins (V&P Scientific, USA) in a 384-format layout.

For the MYTH assay, primer pairs used for bait cloning into pAMBV4 were AMBV_*PEX11*_F/AMBV_*PEX11*_R, AMBV_*MDM10*_F/AMBV_*MDM10*_R and AMBV_*MDM34*_F/AMBV_*MDM34*_R. Primer pairs used for prey cloning into pPR3-N were pPR3N_PEX11_F/pPR3N_PEX11_R, pPR3N_MDM10_F/pPR3N_MDM10_R, pPR3N_MDM12_F/pPR3N_MDM12_R and pPR3N_MDM34_F/pPR3N_MDM34_R. Open reading frames were cloned into plasmids using gap repair homologous recombination [49].

For the BiFC assay, plasmid pFA6a-KANMX6-P_RPL7B_-VN was used for the N-terminal tagging of *PEX11* with the N-terminal part of the Venus fluorescent protein (VN) as previously described [45]. Plasmid pFA6a-VC-HIS3MX6 was used for tagging of *PEX11*, *MDM10* and *MDM34* at their C-terminus with the C-terminal part of Venus (VC) as previously described [34]. The primer pair used for N-terminal tagging was PEX11_N_VN_VC_F/PEX11_N_VN_R and those used for C-terminal tagging were PEX11_C_VC_VN_F/PEX11_C_VC_VN_R, MDM10_C_VC_VN_F/MDM10_C_VC_VN_R and MDM34_C_VC_VN_F/MDM34_C_VC_VN_R. Tags were inserted into the genome of BY4741 by two separate transformations. Correct insertion by homologous recombination was verified by PCR amplification of insert's flanking regions using primers Pex11_c_F/j_KanMX_F (for N-terminal tagging) and j_KanMX_F/Pex11_c_R, j_KanMX_F/Mdm10_c_R and j_KanMX_F/Mdm34_c_R (for C-terminal tagging).

For the peroxisome/ERMES complex co-localization experiment, the strain carrying *MDM34* tagged at its C-terminus with mCherry fluorescent protein was constructed by PCR amplifying the mCherry-CaURA3 cassette from plasmid pFa6a-link-mCherry-CaURA3 using primers MDM34-ChgDNK_F/MDM34-ChgDNK_R and transforming it into the *pex11*Δ Pex14-GFP or corresponding wild-type Pex14-GFP strain. The *pex11*Δ∷*kanMX* Pex14-GFP strain was constructed using pYGFPgN [50] as template and primer pair PEX11_del_F/PEX11_del_R for PCR amplification of the KanMX resistance cassette. Correct insertion into the genome by homologous recombination was verified by PCR amplification of the whole insert's region using primers MDM34_c2_F/MDM34_c_R and PEX11_c_F/PEX11_c_R and of insert's flanking regions using primers MDM34_c2_F/j_KanMX_R, PEX11_c_F/j_KanMX_R and PEX11_c_R/j_KanMX_F.

For Pex11-GFP/Pex3-RFP co-localization studies, the Pex3-RFP strain was crossed to strains *mdm10*Δ, *mdm12*Δ, *mdm34*Δ, *mmm1*Δ and *hst3*Δ and to corresponding wild-type strain all containing Pex11-GFP. Diploids were sporulated and appropriate double fluorescent mutants selected following tetrad dissection.

Genes *MDM12* and *MDM34* were replaced in the Pex25-GFP and Pex27-GFP strains with the KanMX resistance cassette. Primer pairs MDM12_FKO/MDM12_RKO and MDM34_FKO/MDM34_RKO were used for PCR amplification of the cassette from plasmid pKT127 [51]. Correct insertion of the cassette was verified using primer pairs TEFT_F/MDM12_CR and TEFT_F/MDM34_CR.

All primers used for PCR amplification with subsequent transformation and genomic insertion by homologous recombination of the PCR product had approximately 45-nt homology to the desired chromosomal insertion site.

### High-content fluorescence microscopy screen

Imaging of the generated Pex11-GFP collection was performed as described in Wolinski *et al.* [16]. Briefly, 384-format plates were joined 4 by 4 in high-density 1536-format arrays and grown overnight on non-selective YPD medium. Fresh colonies were replicated on 2% agar plates lacking all nutrients and cut in blocks of 8 × 12 colonies. Between individual rows and columns, cuts were made to trap air bubbles and prevent strain cross-contamination. Fluorescence microscopy was performed using a Leica SP2 AOBS confocal microscope (Leica Microsystems, Germany) with spectral detection. We used 63 × oil immersion objective (HCX PL APO NA: 1.32) in combination with 3 × digital zoom. The area covered in each focal plane was 77 μm × 77 μm. Seven optical sections were acquired for each sample with a step of 1 μm, simultaneously for the fluorescent and bright-field channel. GFP fluorescence was excited at 488 nm and emission was recorded at 500–550 nm. Image resolution was 1024 × 1024 pixels. Images are stored in the YPLpex database, which is a derivative of the yeast protein localization database, YPL +^a^, and are available online^4^.

### Object segmentation and extraction of morphological features

Object (peroxisome) segmentation and feature extraction were performed on maximum-intensity projections of GFP channel images using CellProfiler, an open-source software package for image analysis [52]. Peroxisome morphology was summarized with a series of 145 quantitative features describing the distribution of object size, shape and intensity. Collectively, these features define profiles of mutant strains—one real-valued vector per strain—that were used in subsequent computational analysis. Strains with less than 50 or more than 1000 detected objects per image were removed from analysis, since it was likely that profiles of these strains were affected by spurious image segmentation results.

### Preprocessing of morphological profiles

After extracting feature profiles from the Pex11-GFP localization images, we removed features with low variance and eliminated the effects of scale of different features, such that each feature was independently normalized across all growth plates to unit second norm and features whose absolute difference between maximum and minimum value across all plates was below 0.01 were filtered out. After data preprocessing, each strain was described with a profile of 112 features. As the final step, profiles of the reference strain (*yor202w*Δ) that were present in the screen were averaged and the average reference strain was subtracted from each mutant strain. The resulting profiles report on the difference in Pex11-GFP localization patterns between tested mutants and the reference strain.

### Identification of mutants with a specific Pex11-GFP localization pattern

The goal of this task was to identify mutants with abnormal localization patterns that were distinctly different from the localization observed in majority of the mutants. The one-class support vector machines [53] is an established outlier detection algorithm appropriate for this purpose. Outlier detection separated a core of regular mutant profiles, called “inliers”, from those that should be considered different, called “outliers”. This approach is especially useful for high-dimensional data and when the distribution of inlying data cannot be specified. We considered 10% of the mutant strains, 483 strains, with largest distance from the inlier–outlier separating hyperplane. These strains were further prioritized based on their outlyingness score, the distance to the separating hyperplane inferred by the support vector machines algorithm. Intuitively, the further the strain is from the area whose frontier is determined by the separating hyperplane, the more pronounced an outlier it is. Hence, we used the magnitude of the outlyingness score to select outlier strains. The cutoff value used in the outlier criterion was selected by searching for the first kink in the scores that were ordered by their magnitudes in descending order (Supplementary Fig. 1). We assessed the quality of the outlier list with a Kolmogorov–Smirnov test comparing the score distribution of mutant strains to that of the reference strains. Kolmogorov–Smirnov test is a two-sided test for the null hypothesis that two samples are drawn from the same distribution and can be used if sample sizes are different. Further exploratory analyses of outlying mutants were performed in Orange, an open-source data mining and visualization package [24]. The cluster structure was examined by complete-linkage hierarchical clustering with Euclidean distances between outlying mutants. Gene Ontology enrichment [54] was used to link the differences in the Pex11-GFP localization patterns with functional characterization of causal genes/mutations. To characterize the group of mutants with less apparent localization pattern compared to the reference strain, we aimed to find which morphological features of these mutants were substantially different from the inlying strains. For each feature, we computed the Kolmogorov–Smirnov statistic on two samples, where one sample was constructed from feature values of mutants assigned to the group in question and the other sample contained values of the respective feature of inlying strains. Features with high Kolmogorov–Smirnov statistic and low *p*-value indicated specific type of morphology that characterized the group.

### Confocal microscopy, staining and visualization of mitochondria

Unless otherwise noted, all strains were grown to exponential phase in synthetic media and imaged at room temperature on a Leica DMI 6000 B confocal microscope with GFP and Texas Red filter settings.

Wild-type and *pex3*Δ strains containing the Pex11-GFP cassette were grown in YPD medium to exponential phase and stained with MitoTracker Red CMXRos (Life Technologies, USA) for 15 min at 30 °C, washed three times with minimal media and imaged as described above.

Wild-type and *pex11*Δ strains harboring Mdm34-mCherry and Pex14-GFP were imaged using a Leica SP5 confocal microscope (Leica Microsystems, Germany) with spectral detection and a 63 × HCX PL APO NA1.4 oil immersion objective. GFP was excited at 488 nm and emission was detected between 500 and 550 nm. mCherry was excited at 561 nm and emission was detected between 570 and 600 nm. Deconvolution was applied to improve the quality of acquired z-stacks (Huygens 4.0, maximum-likelihood estimation method, 5 iterations; SVI, The Netherlands). Image processing and co-localization analysis was performed using the public domain software Fiji [55]. In brief, processed z-stacks were projected using the maximum-intensity method. Co-localization of GFP and mCherry fluorescence indicated by yellow spots was determined manually in the generated maximum-intensity projections. Co-localization of GFP and mCherry signal was additionally evaluated by interactive inspection of generated multichannel three-dimensional volume rendering representations.

### Western blot and quantitative real-time PCR analyses of Pex11 protein/PEX11 gene expression

To determine Pex11 protein levels in wild-type, *pex3*Δ, *mdm10*Δ, *mdm12*Δ, *mdm34*Δ and *mmm1*Δ deletion strains, we prepared whole cell extracts for immunoblotting from TCA-fixed cells as previously described [56]. Proteins were separated on 10% SDS polyacrylamide gels. After electrotransfer, the nitrocellulose membrane (Bio-Rad) was blocked for 1 h in blocking solution containing 5% dry milk powder in TBST (10 mM Tris–HCl, 150 mM NaCl and 0.05% Tween 20 at pH 8.0). Antibodies used for immunoblotting were anti-GFP-JL-8 (Living Colors, Clontech, France). Ponceau S (0.1% Ponceau S and 5% acetic acid) was used to stain the membrane after transfer as a loading control.

Cultures of the *pex3*Δ*, mdm10*Δ*, mdm12*Δ*, mdm34*Δ and *mmm1*Δ deletion mutants and the corresponding wild-type strain *yor202w*Δ were grown in 100 mL liquid YPD medium at 30 °C to a final OD_600_ of 0.9–1.1, harvested by centrifugation and were washed with 1 mL of nuclease free water before freezing in liquid nitrogen and storing at − 80 °C. RNA was isolated from cells using the PureLink RNA Mini Kit (Ambion, Germany). DNase treatment was performed with the RNase-free DNase kit from Qiagen. The RNA quality and concentration were determined by NanoDrop Spectrophotometer (Thermo Scientific, Germany) and 1% agarose gel electrophoresis. cDNA was synthesized from 0.5 μg RNA using the High-Capacity cDNA Reverse Transcription Kit with RNase Inhibitor (Life Technologies, USA) and random primers according to the manufacturer's manual. *PEX11* expression was monitored by quantitative PCR with the primer pair Pex11-y-50/Pex11-y-51 [57] using also four reference genes, *ALG9, TAF10, TFC1* and *UBC6* [57]. Reactions were performed with LightCycler 480 SYBR Green I Master (Roche Applied Science, Germany) on a Step One Plus Real-Time PCR System (Applied Biosystems). Reactions (5 μL) contained 250 nM of each set of primers and 1.25 ng RNA equivalent cDNA. The initial denaturation was set on 95 °C for 10 min, followed by 45 cycles of 95 °C for 10 s, 62 °C for 10 s and 72 °C for 20 s. A template control for genomic DNA contamination was included in the assay. geNorm analysis for reference genes showed that the average gene expression stability, *M*, ranged from 0.12 (*UBC5* and *TFC1*) to 0.14 (*ALG9*) and the pairwise variation, *V*2/3, among the three most stable genes was 0.03. Relative gene expression was calculated upon normalization to all four reference genes and corrected for primer-specific PCR efficiency as described previously [58].

### MYTH assay

Physical interactions of Pex11 with mitochondrial and cytosolic ERMES complex components (Mdm10, Mdm12 and Mdm34) were tested using the split-ubiquitin-based MYTH system, as previously described [29–32]. Briefly, appropriate pAMBV4-derived bait and pPR3-N-derived prey plasmid pairs were transformed into the NMY51 strain using the lithium acetate method [59]. The NMY51 strains containing bait and prey plasmids were grown in synthetic medium lacking leucine and tryptophan (SD − Leu, − Trp) from an initial OD_600_ of 0.01 for 7–8 generations at 30 °C and were washed with *d*H_2_O before plating 10^5^ cells onto control (SD − Leu, − Trp) and test plates (SD − Leu, − Trp, − Ade, − His, containing 5-bromo-4-chloro-3-indolyl-β,d-galactopyranoside and sodium phosphate) [31]. Plates were incubated for 3–5 days at 30 °C.

### BiFC assay

The BiFC assay was performed as previously described [34].

The following are the supplementary data related this article.

Supplementary Table 1 The list of 483 strains corresponding to 468 genes that were identified as outliers with the computational analysis pipeline of Pex11-GFP localization pattern images. The strains are arranged according to primary and secondary hierarchical clustering distributions. The 109 strains with the most pronounced phenotypic changes relative to the reference strain are highlighted in green.

Supplementary Table 2 Table of all oligonucleotide primers used in this study.

Supplementary data to this article can be found online at http://dx.doi.org/10.1016/j.jmb.2015.03.004.

## Footnotes

4 yplpex.uni-graz.at.

5 http://go.princeton.edu/cgi-bin/GOTermFinder.

6 http://yplp.uni-graz.at.
